# Supplementary material for: Structural variation, functional differentiation and expression characteristics of the AP2/ERF gene family and its response to cold stress and methyl jasmonate in Panax ginseng C.A. Meyer
Source: PLoS One. 2020 Mar 16;15(3):e0226055. doi: 10.1371/journal.pone.0226055 (PMC7075567; doi:10.1371/journal.pone.0226055)
Supplement: S1 Fig — The Venn diagram of the sequence comparison between 397 PgERF transcripts identified in this study (left) and 342 AP2/ERF TFs CDS downloaded from Ginseng Genome Database (right). (PDF) [file pone.0226055.s001.pdf]

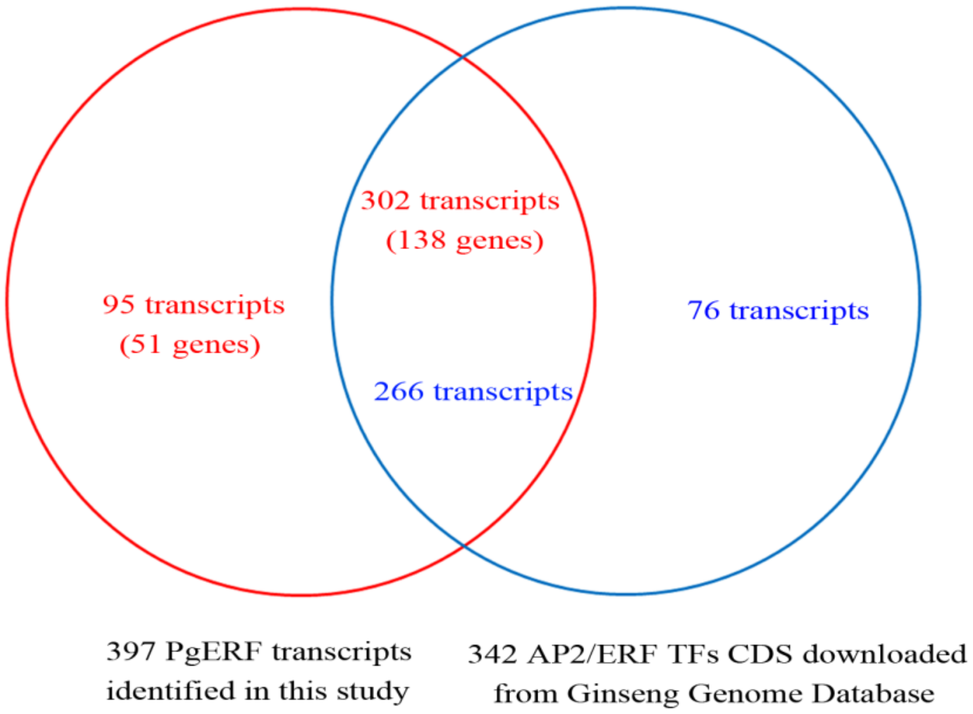

S1 Fig. The Venn diagram of the sequence comparison between 397 *PgERF* transcripts identified in this study (left) and 342 *AP2/ERF* TFs CDS downloaded from Ginseng Genome Database (right).
