# Supplementary material for: Structural variation, functional differentiation and expression characteristics of the AP2/ERF gene family and its response to cold stress and methyl jasmonate in Panax ginseng C.A. Meyer
Source: PLoS One. 2020 Mar 16;15(3):e0226055. doi: 10.1371/journal.pone.0226055 (PMC7075567; doi:10.1371/journal.pone.0226055)
Supplement: S2 Fig — (PDF) [file pone.0226055.s002.pdf]

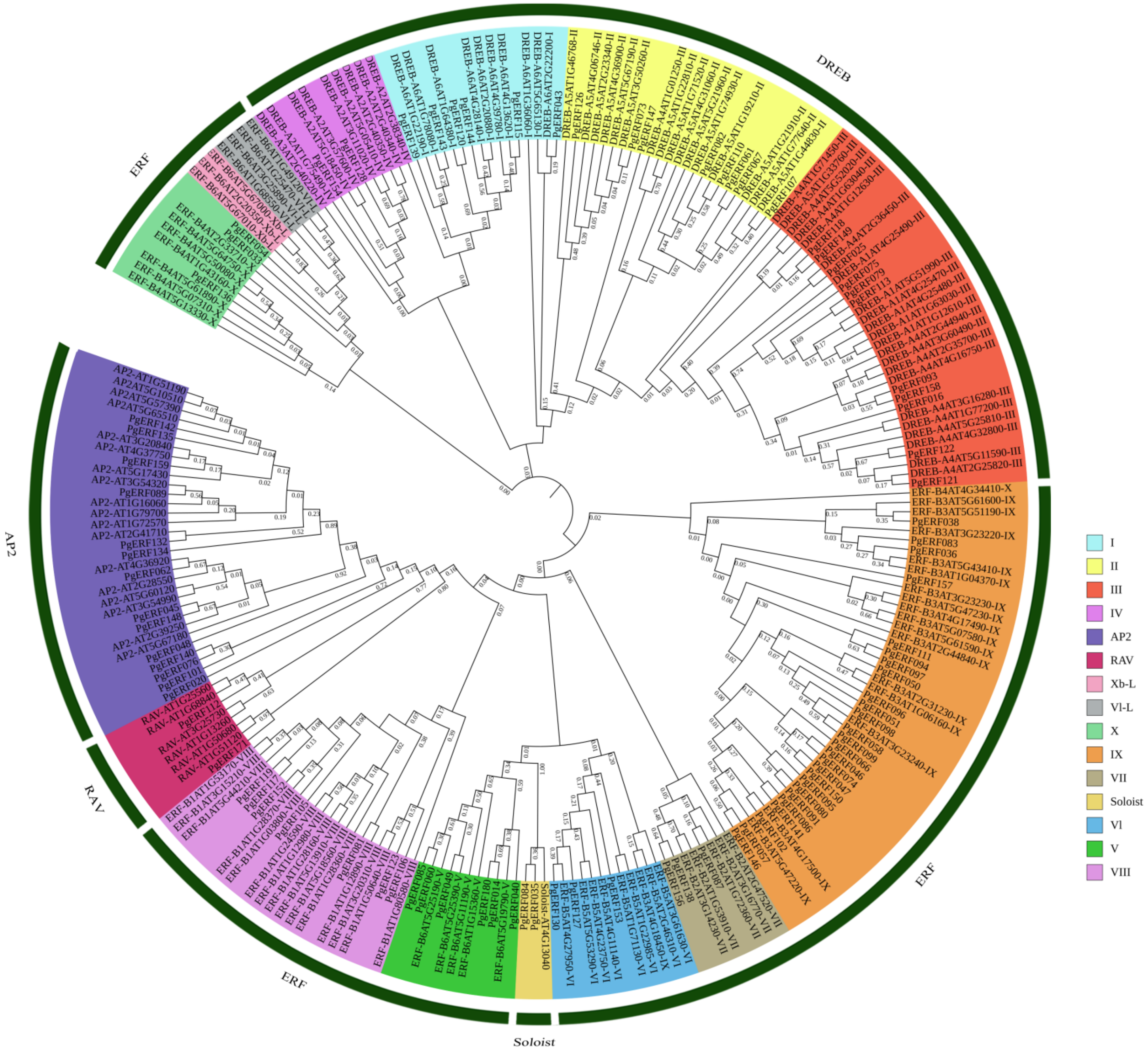

S2 Fig. Phylogenetic tree of the AP2/ERF gene family present in ginseng and Arabidopsis. The amino acid sequences of the AP2 domain were aligned using Clustal W and the phylogenetic tree was constructed using Maximum Likelihood method.
