# Supplementary figures and images for: Structural variation, functional differentiation and expression characteristics of the AP2/ERF gene family and its response to cold stress and methyl jasmonate in Panax ginseng C.A. Meyer
Source: PLoS One. 2020 Mar 16;15(3):e0226055. doi: 10.1371/journal.pone.0226055 (PMC7075567; doi:10.1371/journal.pone.0226055)

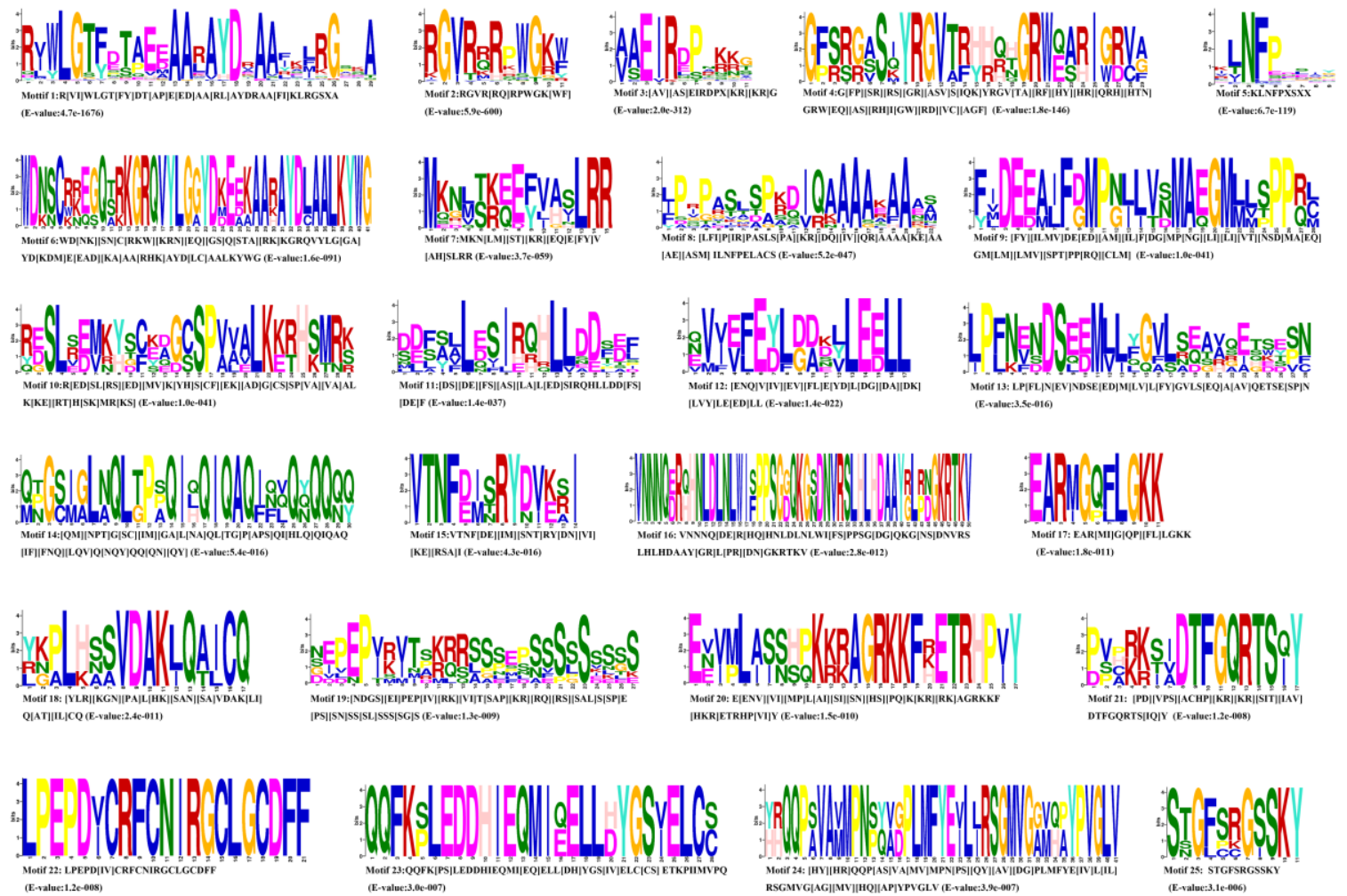

**S3 Fig. Conserved motifs identified from the *PgERF* genes.**

Supplement: S3 Fig — (PDF) [file pone.0226055.s003.pdf]
