# Supplementary material for: Structural variation, functional differentiation and expression characteristics of the AP2/ERF gene family and its response to cold stress and methyl jasmonate in Panax ginseng C.A. Meyer
Source: PLoS One. 2020 Mar 16;15(3):e0226055. doi: 10.1371/journal.pone.0226055 (PMC7075567; doi:10.1371/journal.pone.0226055)
Supplement: S4 Fig — (PDF) [file pone.0226055.s004.pdf]

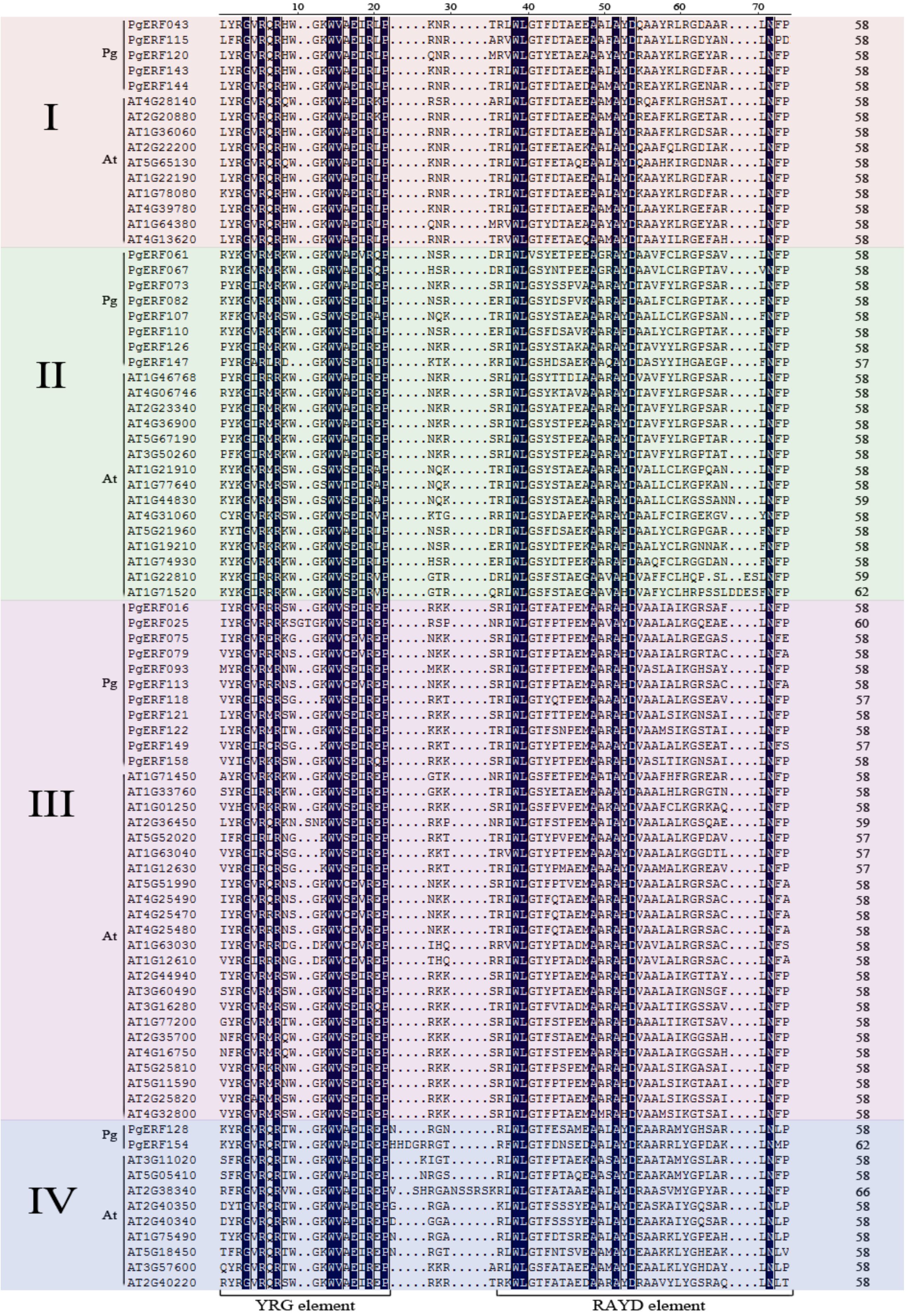

S4 Fig. Comparison of the amino acid sequences of the protein AP2 domains of the DREB subfamily between ginseng and Arabidopsis. The amino acids highlighted in black color represent the conserved amino acid residues, with 100% similarity. Their conserved YRG and RAYD elements are indicated by brackets.
