# Supplementary material for: Structural variation, functional differentiation and expression characteristics of the AP2/ERF gene family and its response to cold stress and methyl jasmonate in Panax ginseng C.A. Meyer
Source: PLoS One. 2020 Mar 16;15(3):e0226055. doi: 10.1371/journal.pone.0226055 (PMC7075567; doi:10.1371/journal.pone.0226055)
Supplement: S5 Fig — (PDF) [file pone.0226055.s005.pdf]

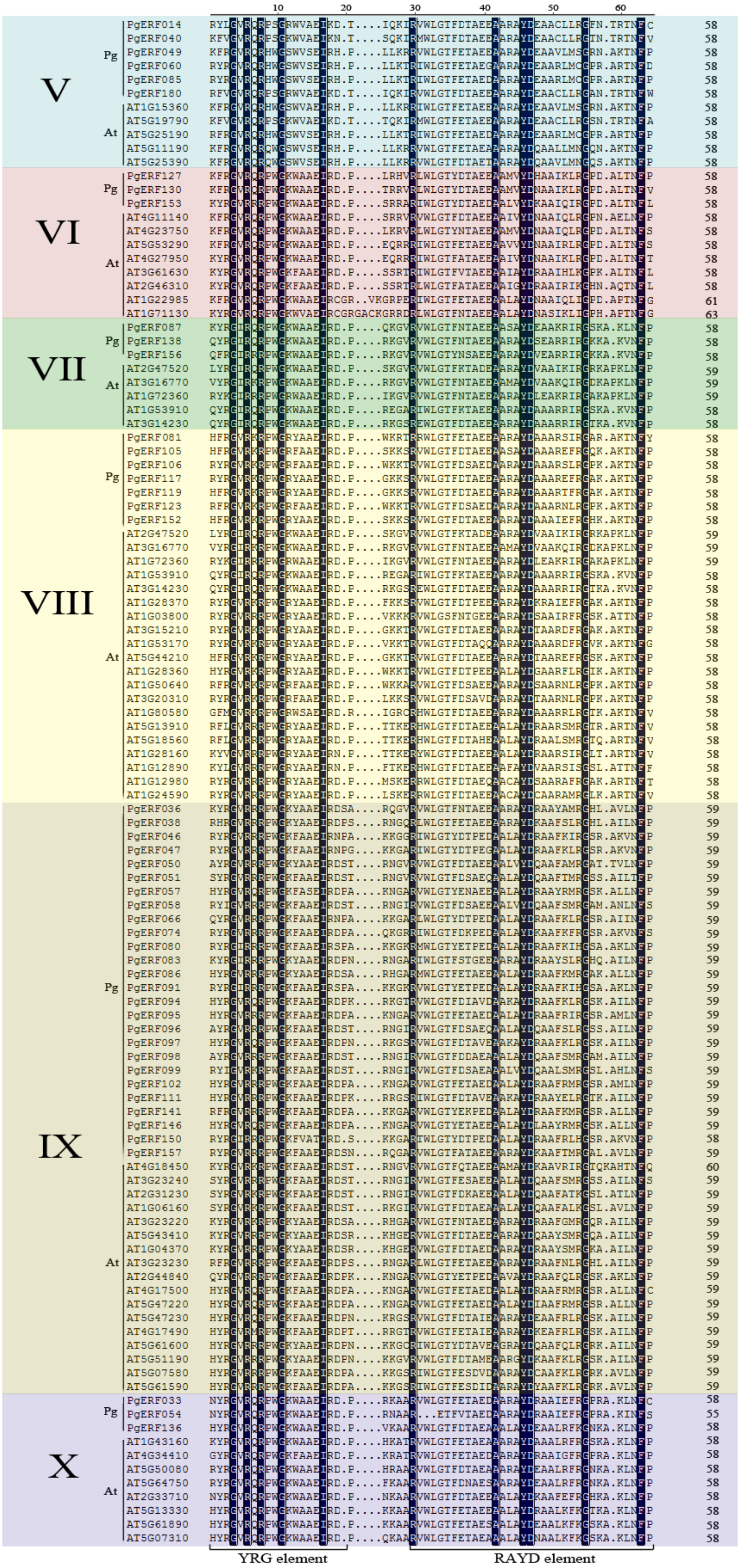

S5 Fig. Comparison of the amino acid sequences of the AP2 domains of the ERF subfamily proteins from ginseng and Arabidopsis. The black background represents the conserved amino acid residues, with 100% similarity. The conserved YRG and RAYD elements are indicated by brackets.
