# Supplementary material for: Structural variation, functional differentiation and expression characteristics of the AP2/ERF gene family and its response to cold stress and methyl jasmonate in Panax ginseng C.A. Meyer
Source: PLoS One. 2020 Mar 16;15(3):e0226055. doi: 10.1371/journal.pone.0226055 (PMC7075567; doi:10.1371/journal.pone.0226055)
Supplement: S6 Fig — (PDF) [file pone.0226055.s006.pdf]

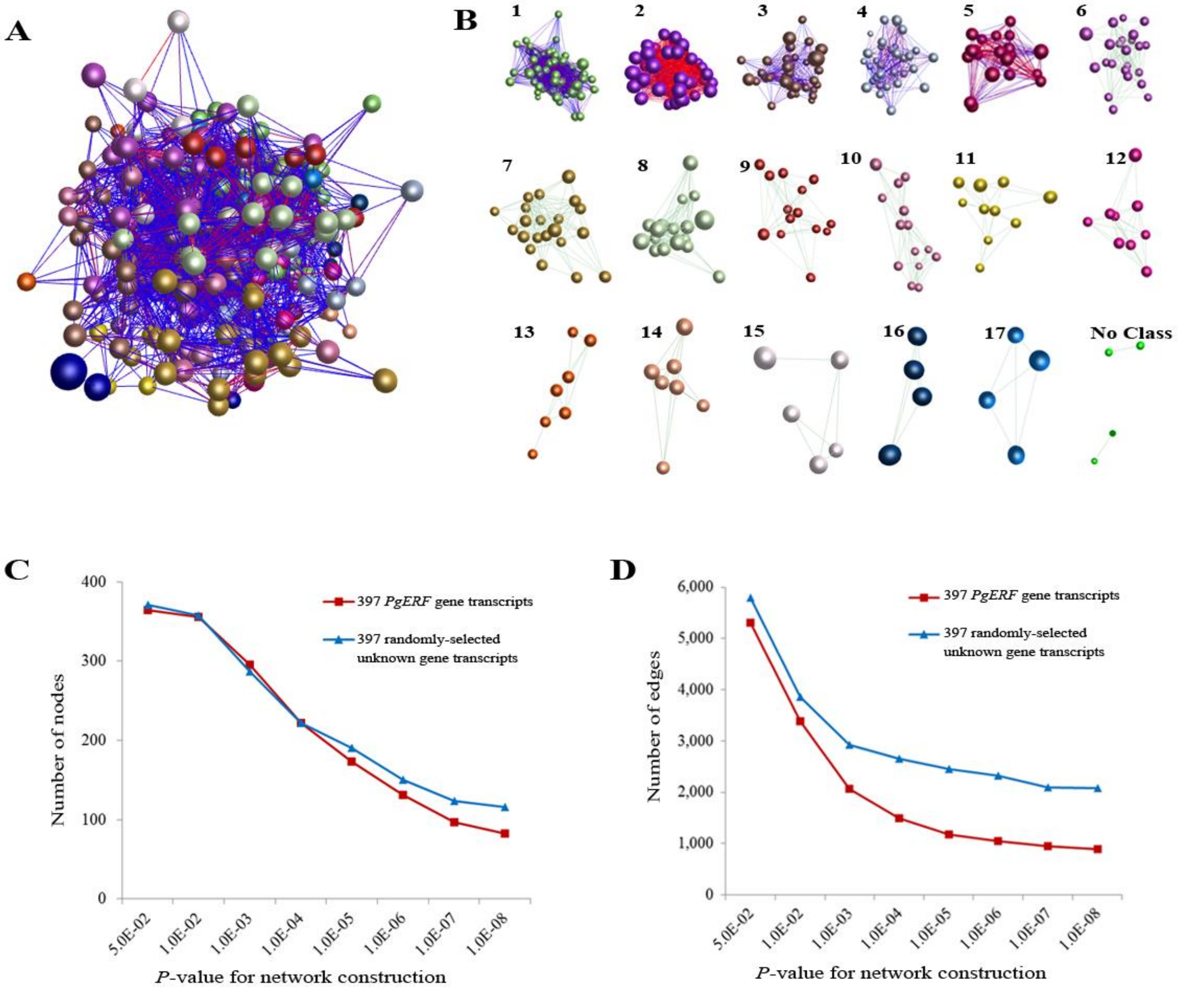

S6 Fig. Co-expression network of the *PgERF* gene transcripts in 14 tissues of a 4-year-old ginseng plant. (A) The co-expression network constructed from 365 of the 397 *PgERF* transcripts at $P \leq 0.05$. (B) 17 clusters of the network. (C) Variation in number of nodes in the network of *PgERF* transcripts at different *P*-values. (D) Variation in number of edges in the network of *PgERF* transcripts at different *P*-values.
